# Supplementary material for: Identification of photoperiod-induced specific miRNAs in the adrenal glands of Sunite sheep (Ovis aries)
Source: Front Vet Sci. 2022 Jul 22;9:888207. doi: 10.3389/fvets.2022.888207 (PMC9354845; doi:10.3389/fvets.2022.888207)
Supplement: Supplementary file 1 [file Data_Sheet_1.ZIP › Supplementary file/miRDeep2 file.zip!/miRDeep2 file/Know_Structure/LP42A2/oar-mir-329b.pdf]

miRBase precursor : oar-mir-329b
Total read count : 1428
oar-miR-329b-5p read count : 20
oar-miR-329b-3p read count : 1394
remaining reads : 14

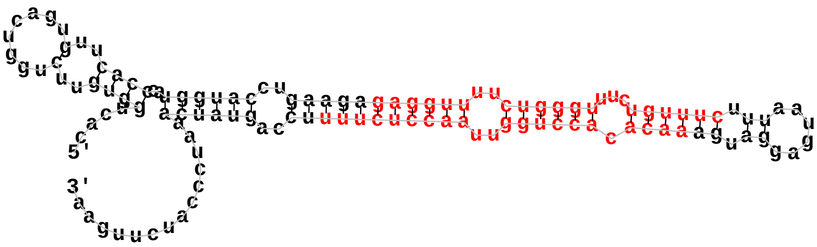

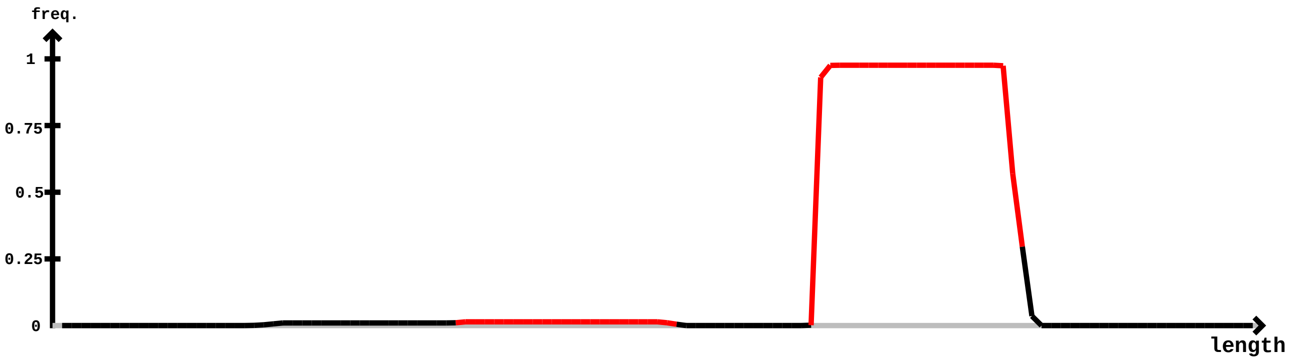


```
                                                                                          oar-miR-329b-3p
                                        oar-miR-329b-5p
5'- cacugguguucuggucaguguucacccaugguaccugaagagagguuuucuggguuucuguuucuuuaaugaggaugaaacacaccugguuaaccucuuuuccaguaucaaaucccaucuugaa  -3'  exp
    ....((((..(........)..))))..((((((..(((((((((((..((((((...((((((.((......)).)))))).))))))..)))))))))))..))))))..............    reads   mm
    ...................guucacccaugguaccugaag....................................................................................    1       0
    ....................uucacccaugguaccugaaga...................................................................................    3       0
    .....................ucacccaugguaccugaaga...................................................................................    5       0
    ......................cacccaugguaccugaaga...................................................................................    5       0
    ........................................agagguuuucuggguuucuguu..............................................................    2       0
    .........................................gagguuuucuggguuucuguu..............................................................    3       0
    .........................................gagguuuucuggguuucuguuu.............................................................    8       0
    .........................................gagguuuucuggguuucuguuuc............................................................    7       0
    .............................................................................aaacacaccugguuaaccucu..........................    2       0
    ..............................................................................aacacaccugguuaaccuc...........................    3       0
    ..............................................................................aacacaccugguuaaccucu..........................    573     0
    ..............................................................................aacacaccugguuaaccucuu.........................    386     0
    ..............................................................................aacacaccugguuaaccucuuu........................    349     0
    ..............................................................................aacacaccugguuaaccucuuuu.......................    16      0
    ...............................................................................acacaccugguuaaccucu..........................    3       0
    ...............................................................................acacaccugguuaaccucuu.........................    4       0
    ...............................................................................acacaccugguuaaccucuuu........................    22      0
    ...............................................................................acacaccugguuaaccucuuuu.......................    35      0
    ................................................................................cacaccugguuaaccucuu.........................    1       0
```
